# A novel method for rapid detection of a *Helicobacter pylori* infection using a γ-glutamyltranspeptidase-activatable fluorescent probe

**DOI:** 10.1038/s41598-019-45768-x

**Published:** 2019-07-01

**Authors:** Taro Akashi, Hajime Isomoto, Kayoko Matsushima, Mako Kamiya, Tsutomu Kanda, Masayuki Nakano, Takumi Onoyama, Masashi Fujii, Junko Akada, Yuko Akazawa, Ken Ohnita, Fuminao Takeshima, Kazuhiko Nakao, Yasuteru Urano

**Affiliations:** 1 Department of Gastroenterology and Hepatology, Nagasaki University Graduate School of Biomedical Sciences, 1-7-1 Sakamoto, Nagasaki, 852-8501 Japan; 2 Divison of Medicine and Clinical Science, Faculty of Medicine, Tottori University, 36-1 Nishi-cho, Yonago, 683-8504 Japan; 3 Graduate School of Medicine, The University of Tokyo, 7-3-1 Hongo, Bunkyo-ku, Tokyo 113-0033 Japan; 4 Graduate School of Pharmaceutical Sciences, The University of Tokyo, 7-3-1 Hongo, Bunkyo-ku, Tokyo 113-0033 Japan; 5 Department of Bacteriology, Institute of Tropical Medicine, Nagasaki University, Nagasaki, Japan; 6 Department of International Health, Institute of Tropical Medicine, Nagasaki University, Nagasaki, Japan; 7 Department of Environmental and Preventive Medicine, Oita University Faculty of Medicine, Yufu, Japan

**Keywords:** Fluorescence imaging, Gastritis

## Abstract

A γ-glutamyl hydroxymethyl rhodamine green probe (gGlu-HMRG) reacts with γ-glutamyltranspeptidase (GGT) and immediately produces fluorescence, is clinically applied for real-time cancers’ visualization. Since *Helicobacter pylori* produces GGT, this study aimed to investigate whether gGlu-HMRG can be used to detect *H. pylori* infections. A wild-type *H. pylori* strain and the *ggt* gene-disrupted mutant were cultured and treated with gGlu-HMRG. This fluorescent probe assay was used to quantify GGT activity of *H. pylori ex vivo* using gastric biopsy specimens. The *H. pylori* diagnostic capabilities of the assay were determined from altered fluorescence intensity (FI) values at 5 min (FIV-5) and 15 minutes (FIV-15). Distinct fluorescence was identified in wild *H. pylori* strain, using gGlu-HMRG, whereas no fluorescence was observed in *ggt* gene-disrupted mutant strain. On *ex vivo* imaging of gGlu-HMRG, fluorescence intensity increased markedly with time in *H. pylori*-positive specimens; however, the *H. pylori*-negative specimens displayed a slight increase in FI. FIV-5 and FIV-15 differed significantly between *H. pylori*-positive and -negative specimens. FIV-15 differed significantly between *H. pylori*-positive and -eradicated group. This assay sensitivity and specificity were 75.0% and 83.3% in the antrum and 82.6% and 89.5% in the stomach body. GGT-activatable fluorescence probe is applicable for rapid diagnosis of *H. pylori*.

## Introduction

γ-glutamyltranspeptidase (GGT) is a prototypical hepatic enzyme; however, it is expressed on the plasma membranes of most cells^1^. GGT is notably overexpressed in cells and tissues in various cancers, engaging in cellular glutathione homeostasis and promoting tumor progression and invasion and chemotherapeutic resistance^2^.

*Helicobacter pylori* infections are one of the most common worldwide, inducing chronic gastric mucosal inflammation, atrophy, and intestinal metaplasia; ultimately, some individuals with chronic infection develop gastric cancer^3^. Thus, accurate diagnosis of this infection is clinically critical. *H. pylori* reportedly produces conservative GGT^4^. *H. pylori* GGT converts extracellular glutamine and glutathione to glutamate and transports it into host cells, wherein glutamate is incorporated into the tricarboxylic acid cycle or used for glutamine synthesis^5^. Consequently, *H. pylori* GGT hence deprives cells of extracellular glutamine and glutathione, produces ammonia, and increases reactive oxygen species levels^4^. GGT facilitates gastric colonization of *H. pylori*^6^, induces apoptosis in gastric epithelial cells^4^, and inhibits T-cell proliferation and dendritic cell differentiation^7–10^. Thus, GGT plays an important role in the pathogenesis of *H. pylori* infections, resulting in gastric mucosal injuries. Moreover, a previous study reported a significant increase in GGT activity of *H. pylori* isolates obtained from individuals with peptic ulcers than in those with non-ulcer dyspepsia^11^.

Urano *et al*. developed an enzymatically activatable fluorescent probe, γ-glutamyl hydroxymethyl rhodamine green (gGlu-HMRG), which is non-fluorescent under a normal cellular environment but becomes highly fluorescent upon enzymatic catalysis of GGT^12^. This enhanced GGT in cells and tissues in various cancers potentially reacts with gGlu-HMRG and emits strong fluorescence for a rather short duration^13,14^. Thus, the gGlu-HMRG assay is a potentially novel cancer diagnostic method. Owing to the hydrophobic nature of HMRG, it rapidly penetrates the plasma membrane, subsequently accumulating primarily in intracellular lysosomes and enhancing fluorescence specifically in enzyme-expressing cells^12^.

This study aimed to investigate whether gGlu-HMRG emits fluorescence upon reacting with GGT produced by *H. pylori*. An *ex vivo* imaging assay for GGT activity with gGlu-HMRG was conducted to detect *H. pylori* infection using clinical samples.

## Methods

### Reagents

gGlu-HMRG, an activatable fluorescent imaging probe, was synthesized as previously described^12^. A 10-mM stock solution of gGlu-HMRG was stored at −20 °C until use. A 10-mM solution of gGlu-HMRG was thawed at room temperature and diluted to 50 μM in phosphate-buffered saline (PBS)^12^.

### *In vitro* gGlu-HMRG imaging analyses using laboratory *H. pylori* strains

*H. pylori* strain 26695 was used as a standard strain in this study. The *ggt* gene-disrupted mutant of *H. pylori* strain 26695 was kindly provided by Dr. Keigo Shibayama (National Institute of Infectious Diseases, Tokyo, Japan). *H. pylori* strains were cultured in Brucella broth (BD, Franklin Lakes, NJ, USA) supplemented with 10% fetal bovine serum at 37 °C for 48 h with agitation under microanaerobic conditions and washed with PBS, and a 10^8^ cells/ml bacterial suspension was prepared in PBS.

To carry out the reaction with gGlu-HMRG *in vitro*, *H. pylori* suspensions of both the wild-type and the *ggt* gene-disrupted mutant strains were serially diluted 10-fold with PBS as indicated, i.e., 10 to 10^8^ cells/ml. gGlu-HMRG was also diluted with PBS and adjusted to 50 μM. Thereafter, 16 μl of each diluted *H. pylori* strain was placed in strips of 8 tubes and treated with 4 μl of prepared gGlu-HMRG. Fluorescence excitation was quantified for 15 min as reported previsouly^15^. To carry out the reaction with GGT inhibitor *in vitro*, *H. pylori* suspensions were centrifuged and separated into supernatant and pellet fractions. The pellet fraction was remixed with the same amount of PBS. Sixteen microliters of supernatant or remixed pellet was added to 4 μl of gGlu-HMRG adjusted to 50 μM, or 4 μl of a solution of gGlu-HMRG adjusted to 50 μM plus GGT inhibitor adjusted to 250 μM and observed for 10 min. GGsTop (ako Pure Chemical Industries, Ltd, Osaka, Japan) was used as the GGT inhibitor. *In vitro* experiments were performed at room temperature.

### Human gastric samples

Patients (n = 46; 31 men, 15 women; mean age, 63.8 ± 8.85 and 70.1 ± 12.4 years, respectively) who underwent esophagogastroduodenoscopy were enrolled in this study. The Nagasaki University Hospital ethics committee approved the study (#15012681) and patients provided written informed consent to participate in the study. Patients were considered *H. pylori*-positive when either the urea breath test or rapid urease test or both tests yielded positive outcome. On the other hand patients were considered *H. pylori*-negative when both the urea breath tests and rapid urease tests yielded negative outcome. As a whole, there were 21 *H. pylori*-positive cases (45.7%) and 25 *H. pylori*-negative cases (54.3%). Nevertheless, in cases of severe gastric atrophy, even considered *H. pylori*-negative via the urea breath and rapid urease testing, spontaneous eradication may occur owing to severe atrophy accompanied by extensive intestinal metaplasia; hence, such cases were further assessed via the stool antigen test, which revealed negative outcome in each case, thus deeming such 4 patients *H. pylori*-negative. Four cases underwent eradication therapy and successful eradication was confirmed at least one month after eradication therapy, the duration after eradication ranging 11 months to 6 years. As the definition, *H. pylori*-negative group included 17 *H. pylori*-infection-free cases and 4 possibly *H. pylori*-spontaneous-eradication cases, discriminating from the really *H. pylori*-eradicated group via successful eradication therapy (4 *H. pylori*-eradication-success cases). Collectively, the patients enrolled in this study were divided into following the 3 groups; the *H. pylori*-positive group (n = 21), the *H. pylori*-negative group (n = 21) and the *H. pylori*-eradicated group (n = 4). Separate biopsy samples were harvested from the antrum and the stomach body of each patient and rinsed with PBS. A neutral pH was confirmed using a litmus paper. The samples were subjected to *ex vivo* imaging tests for gGlu-HMRG.

### *Ex vivo* gGlu-HMRG imaging tests using stomach specimens

Each stomach specimen was treated with 20 μl of gGlu-HMRG adjusted to 50 μM, placed in the dark, and exposed to light at an excitation wavelength of 470 nm at room temperature (Limited-STAGE, ALB-470, AMZ, Osaka, Japan). The specimens were photographed using a commercially available digital camera at 1, 5, and 15 min after initial exposure to gGlu-HMRG. Fluorescence intensities (FI) were quantified in the photographed images, using Image J 1.50 (National Institutes of Health, Bethesda, MD, USA)^12^. The values obtained by subtracting the FI 5 min after treatment with gGlu-HMRG from FI at 5 (defined as FIV-5) and 15 minutes (defined as FIV-15) were used to assess the diagnostic capability of the assay to detect *H. pylori* infection.

### *H. pylori*/GGT double-immunofluorescence staining

Stomach biopsy specimens (antrum and stomach body) of *H. pylori*-positive patients were fixed with formalin, embedded in paraffin, and sliced into 4-μm-thick sections. Tissue sections were deparaffinized and antigen retrieval was performed via heat treatment with a Target Retrieval Solution (DAKO North America, Inc., Carponteria, CA, USA) diluted with distilled water. Thereafter, sections were incubated with Protein Block Serum Free solution (DAKO North America, Inc.) at room temperature for 20 min. An anti-*H. pylori* rabbit polyclonal antibody (DAKO North America, Inc.; 1:150) and a mouse monoclonal anti-human GGT antibody (Abnova, Taipei, Taiwan; 1:150) were applied as primary antibodies and incubated with the tissue sections in a humidified chamber overnight at 4 °C. The slide was washed thrice in PBS with Tween-20 (PBS-T) for 5 min each. Thereafter, Alexa Fluor 488 goat anti-rabbit IgG (Invitrogen, Carlsbad, CA, USA; 1:200) and Alexa Fluor 568 goat anti-mouse IgG (Invitrogen; 1:200) were applied as secondary antibodies and incubated with the tissue sections for 1 h at room temperature in the dark. The slide was washed thrice in PBS-T for 5 min each. Fluorescence microscopic images were obtained using an All-in-One Fluorescence Microscope (BZ-X700; KEYENCE Japan, Osaka, Japan), and images were captured at × 1000 magnification.

### Statistical analysis

Statistical analysis was performed using JMP Pro 13.0.0 for Macintosh OS (SAS Institute, Inc., Cary, NC, USA). The Wilcoxon rank-sum test was performed to compare the mean values between 3 groups (*H. pylori-*positive vs. *H. pylori*-negative vs. *H. pylori*-eradicated one). The receiver operating characteristic (ROC) curve was obtained from logistic regression analysis, and the optimum cutoff FIVs were determined using the Youden Index. Logistic regression analysis was performed to investigate the association between the results of gGlu-HMRG assay and the Kyoto classification of gastritis^3^. Variables for logistic regression analysis were selected in a stepwise manner. A p value of < 0.05 was considered statistically significant.

## Results

### GGT activity assessed using gGlu-HMRG in laboratory *H. pylori* strains

**Figure 1:**
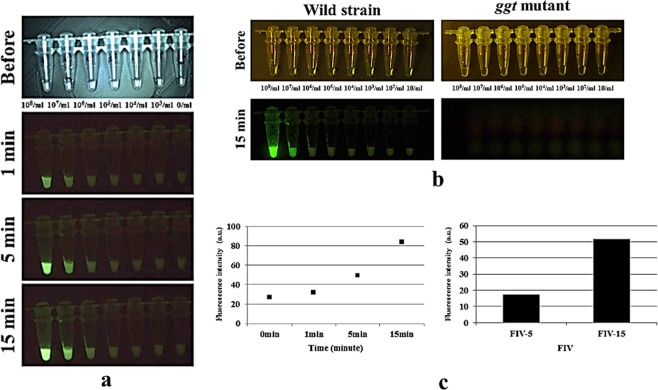
(**a**) The *H. pylori s*train 26695 and the *ggt* gene-disrupted mutant strain were cultured and reacted with 50 μM of γ-glutamyl hydroxymethyl rhodamine green probe (gGlu-HRMG) in phosphate-buffered saline (PBS). Experiments were performed at densities ranging from 10 cells/ml to 10^8^ cells/ml. The wild-type *H. pylori* strain emitted green fluorescence over time. (**b**) The *ggt*-gene disrupted mutant strain emitted nil fluorescence upon gGlu-HMRG exposure for 15 min, even at high cell densities. (**c**) Fluorescence intensity (FI) remained unchanged at 1 min. FI increased at 5 and more distinctly at 15 min, displaying substantial alterations in FIV-5 and FIV-15; values obtained by subtracting FI 5 min after treatment with gGlu-HMRG, FIV-15; FI at 15 min after treatment with gGlu-HMRG, respectively, are observed.

**Figure 2:**
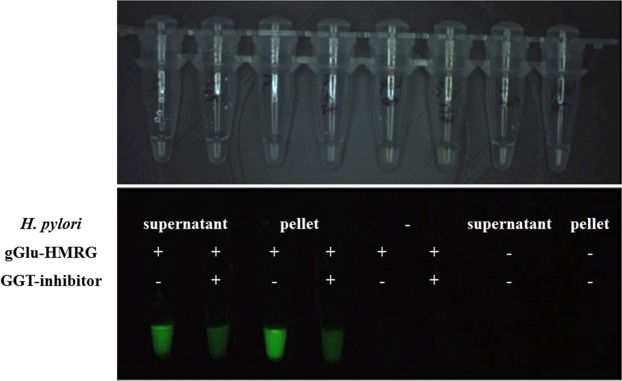
The wild-type laboratory strain was centrifuged and separated into supernatant and pellet fractions. Each fraction was treated with 50 μM of gGlu-HMRG with or without an inhibitor of γ-glutamyltranspeptidase (GGT). Addition of the GGT inhibitor suppressed green fluorescence by the wild-type strain. Fluorescence was observed in both fractions, albeit at a greater intensity in the former.

We investigated whether gGlu-HMRG was activated by GGT produced by *H. pylori* cells *in vitro*. Experiments were performed using both the wild-type and the *ggt*-gene disrupted mutant strains at densities ranging from 10 to 10^8^ cells/ml. Both strains were treated with gGlu-HMRG, and fluorescence was observed for 15 min. Wild-type *H. pylori* emitted fluorescence; the FI tended to increase with an increase in cell density (Fig. 1a). At low cell densities, even the wild-type *H. pylori* strain emitted slight fluorescence upon exposure to gGlu-HMRG. In contrast, the *ggt*-gene disrupted mutant strain did not emit fluorescence upon gGlu-HMRG exposure even at high cell densities (Fig. 1b). Fluorescence emitted immediately after probe addition and the FI remained unchanged at 1 min. However, FI increased at 5 min and more distinctly at 15 min (Fig. 1a,c), displaying substantial alterations in FIV-5 and FIV-15. Notably, treatment with the GGT inhibitor suppressed fluorescence emission by the wild-type strain (Fig. 2). Upon centrifugation of the suspension, green fluorescence was again detected both in the bacterial pellet and the supernatant, albeit slightly greater in the former (Fig. 2).

### *Ex vivo* imaging analysis of gGlu-HMRG using stomach biopsy specimens

**Figure 3:**
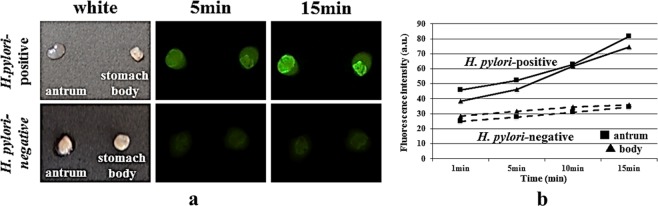
(**a**) Gastric biopsy specimens were treated with 50 μM gGlu-HMRG, one obtained from the antrum and the other from the stomach body. In specimens obtained from a *H. pylori*-positive patient, the fluorescent signal was immediately detected no more than 5 min after treatment with gGlu-HMRG, and strong fluorescence was emitted both in antral and stomach body specimens. However, in specimens from obtained from a *H. pylori*-negative patient, the fluorescence was rather weak, irrespective of sampling sites. (**b**) Fluorescence intensity increased with time, increasing substantially at 15 min after gGlu-HMRG treatment and increasing slightly in *H. pylori*-negative specimens.

**Figure 4:**
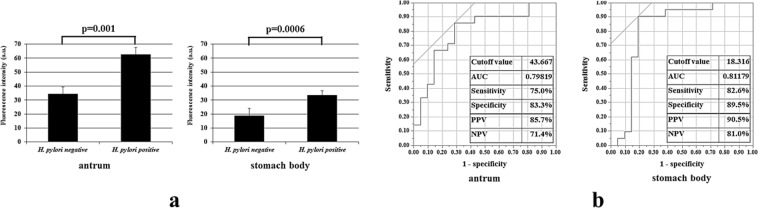
(**a**) The values obtained by subtracting FI at 1 min after treatment with gGlu-HMRG from FI at 15 min (defined as FIV-15) were compared between the *H. pylori*-negative and the *H.pylori*–positive group. There were significant differences in FIV-15 between the 2 groups both in antral and the stomach body specimens. (**b**) The optimum threshold for FIV-15 was determined for the antrum and stomach body via receiver operating characteristic curve analysis. AUC: Area under the Curve, PPV: Positive Predictive Value, NPV: Negative Predictive Value.

**Figure 5:**
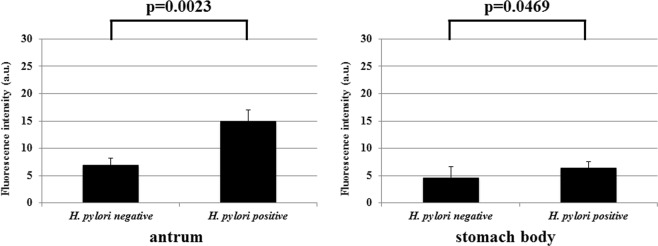
The values obtained by subtracting FI at 1 min after treatment with gGlu-HMRG from FI at 5 min (defined as FIV-5) were compared between the *H. pylori*-negative group and the *H.pylori*-positive patients. There were significant differences in FIV-5 between the 2 groups in both antral and the stomach body specimens.

Two separate stomach biopsy specimens (the antrum and the stomach body) from each patient were treated with gGlu-HMRG. FI was determined over time (at 1, 5, and 15 min). In specimens obtained from the representative *H. pylori*-positive patients, the fluorescent signal was immediately detected no more than 5 min after gGlu-HMRG treatment (Fig. 3a), and FI increased with time in both the antrum and stomach body specimens (Fig. 3b). However, in specimens obtained from *H. pylori*-negative patients, the FI was rather weak, irrespective of the sampling sites (Fig. 3a), displaying only a slight but not significant elevation in FI (Fig. 3b). Furthermore, FIV-15 differed significantly between the *H. pylori*-positive and the *H. pylori*-negative group either in antral specimens (p = 0.001) or in the stomach body specimens (p = 0.0006) (Fig. 4a). Upon ROC analysis, the cut-off FIV-15 was at 43.667 and 18.316, for antral specimens and for the stomach body specimens, respectively (Fig. 4b). The sensitivity and specificity of *ex vivo H. pylori* GGT-activatable fluorescence assay were 75.0% (18/24) and 83.3% (15/18), respectively, for antral specimens, and 82.6% (19/23) and 89.5% (17/19), respectively, for the stomach body specimens (Fig. 4b). The positive and negative predicted values for the assessment of *H. pylori* status were 85.7% (18/21) and 71.4% (15/21), respectively, for antral specimens, and 90.5% (19/21) and 81.0% (17/21), respectively, for the stomach body specimens. Again, FIV-5 differed significantly between the *H. pylori*-positive and the *H. pylori*-negative group either in antral specimens (p = 0.0023) or in the stomach body specimens (p = 0.0469) (Fig. 5).

**Figure 6:**
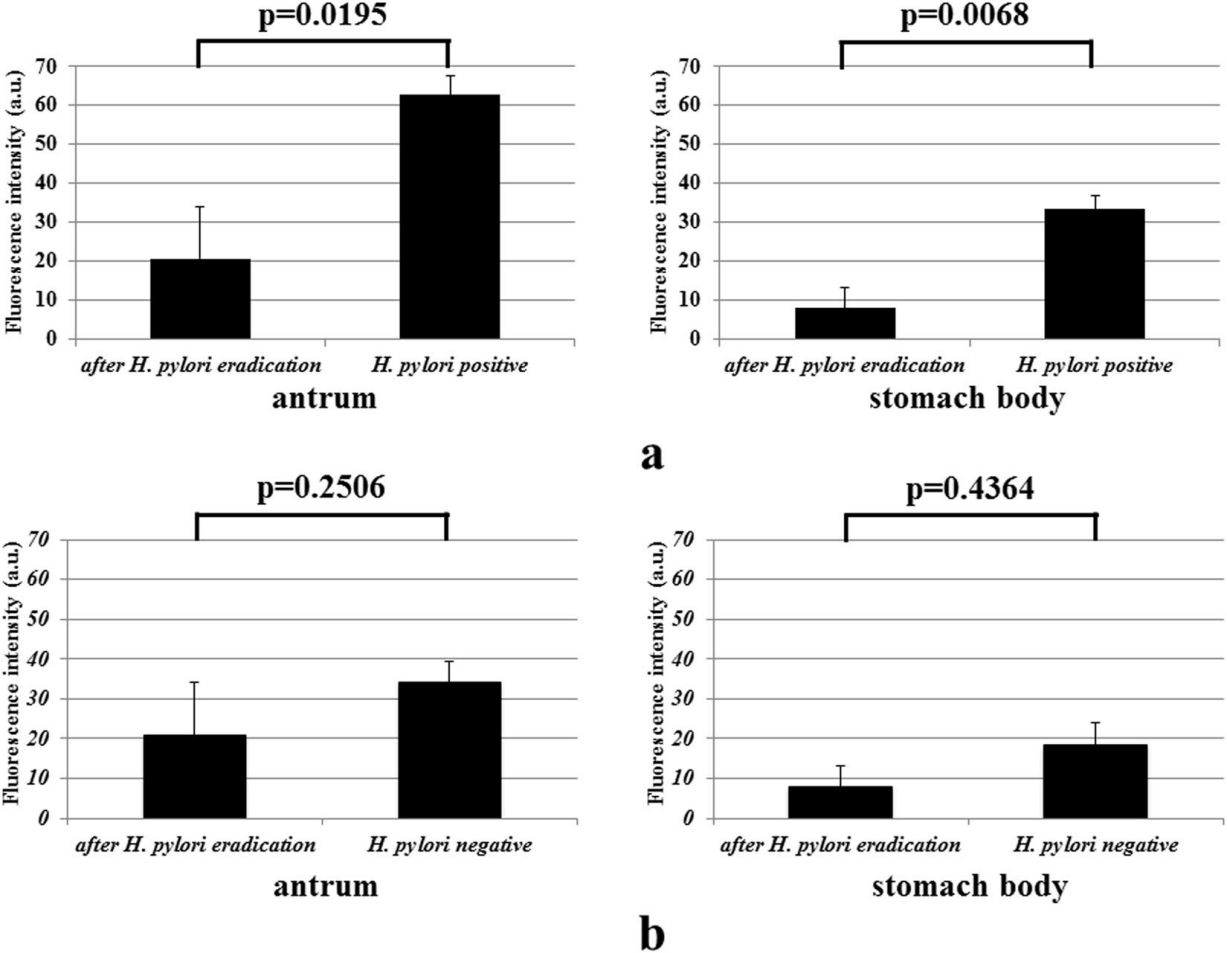
(**a**) The FIV-15 of specimens obtained from the *H. pylori-*positive group were significantly greater than the FI of specimens obtained from the *H. pylori-*eradicated group upon assessment of antral (left panel) and the stomach body (right panel) specimens. (**b**) There were no significant FIV-15 between the *H. pylori*-eradicated group and the *H. pylori*-negative group.

FIV-15 differed significantly between the *H. pylori*-positive and the *H. pylori*-eradicated group either in antral specimens (p = 0.0195) or in the stomach body specimens (p = 0.0068) (Fig. 6a). However, the FIV-15 did not differed significantly between the *H. pylori*-negative and the *H. pylori*-eradicated group either in antral specimens (p = 0.2506) or in the stomach body specimens (p = 0.4364) (Fig. 6b).

### Co-localization of *H. pylori* and GGT in stomach biopsy specimens via double-immunofluorescence staining

**Figure 7:**
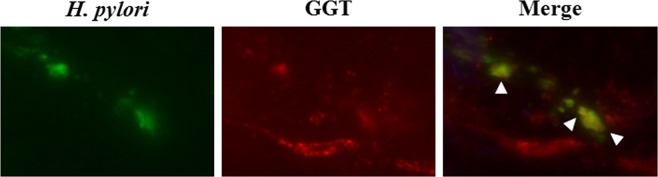
Double-immunofluorescence staining was performed to assess the expression of *H. pylori* GGT in *H. pylori*-positive stomach biopsy specimens. Using the *H. pylori*-positive antral biopsy specimens, *H. pylori* fluorescence (green fluorescence) and GGT (red) were detected. Co-localization between portions of *H. pylori* and GGT is shown using arrow heads.

Immunofluorescence staining was performed to visualize the expression of *H. pylori* GGT in *H. pylori*-positive stomach biopsy specimens. Using the *H. pylori*-positive antral biopsy specimens, *H. pylori* fluorescence (green fluorescence) and GGT (red) were observed. Co-localization of *H. pylori* and GGT is shown in Fig. 7.

### Correlation between *H. pylori* infection status via the *ex vivo* gGlu-HMRG imaging assay and Kyoto classification of gastritis

*H. pylori* infection and chronic gastritis are reportedly closely associated, and the Kyoto classification of gastritis is used to diagnose *H. pylori* infection from endoscopic findings^3^. The association between the results of the gGlu-HMRG assay and endoscopic findings in accordance with the Kyoto classification of gastritis were assessed via univariate and multivariate analyses. On univariate analysis, the *H. pylori* infection status assessed through fluorescence exhibited significant correlations with *H. pylori* infection status and atrophy, while the *H. pylori* infection status assessed via the gGlu-HMRG imaging analysis correlated significantly only with *H. pylori* infection status assessed via multivariate analysis (Table 1, the antrum). Furthermore, the *H. pylori* infection status assessed via gGlu-HMRG, using stomach body specimens, exhibited significant correlations with the *H. pylori* infection status, atrophy, and diffuse redness upon univariate analysis. *H. pylori* infection status assessed via gGlu-HMRG imaging analysis and that assessed via multivariate analysis displayed a significant correlation (Table 2, the stomach body). Furthermore, upon *ex vivo* gGlu-HMRG imaging, significant differences (p < 0.05) were observed in the extent of atrophic gastritis per the thresholds in both antral and the stomach body (Supplemental Information).

**Table 1:** Association between the results of the *ex vivo* gGlu-HMRG assay in the antrum and endoscopic findings of the Kyoto classification of gastritis.

Factor	Univariate analysis			Multivariate analysis		
	OR*	95%CI**	*P*	OR*	95%CI**	*P*
*H. pylori* infection status	15.00	3.20 ~ 70.39	0.0006	8.00	1.16 ~ 55.26	0.0349
Atrophy	11.00	2.37 ~ 51.14	0.0022	2.75	0.38 ~ 19.67	0.3136
Intestinal metaplasia	2.06	0.45 ~ 9.42	0.3519			
Diffuse redness	8.00	1.50–42.65	0.0149	1.67	0.20–14.03	0.6376
Hypertrophy of gastric fold	1.75	0.43 ~ 7.08	0.4328			
Nodularity	—	—	—			
Foveolar-hyperplastic polyp	—	—	—			
Xanthoma	0.70	0.15 ~ 3.28	0.6509			
Sticky mucus	8.50	0.95 ~ 75.80	0.0552			
Regular arrangement of collecting venules	0.33	0.08 ~ 1.27	0.1077			
Fundic gland polyp	—	—	—			
Red streak	—	—	—			

^*^Odds ratio.

^**^Confidence interval.

**Table 2:** Association between the results of the *ex vivo* gGlu-HMRG assay in the stomach body and endoscopic findings of the Kyoto classification of gastritis.

Factor	Univariate analysis			Multivariate analysis		
	OR*	95%CI**	*P*	OR*	95%CI**	*P*
*H. pylori* infection status	40.38	6.55 ~ 248.98	<0.0001	24.54	2.53 ~ 238.19	0.0058
Atrophy	14.44	3.06 ~ 68.18	0.0007	1.08	0.09 ~ 13.54	0.9505
Intestinal metaplasia	4.53	0.83 ~ 24.76	0.081			
Diffuse redness	4.89	1.11 ~ 21.47	0.0356	2.54	0.22 ~ 29.24	0.4541
Hypertrophy of gastric fold	2.00	0.49 ~ 8.09	0.331			
Nodularity	—	—	—			
Foveolar-hyperplastic polyp	—	—	—			
Xanthoma	0.79	0.17 ~ 3.69	0.7639			
Sticky mucus	1.88	0.40 ~ 8.82	0.4223			
Regular arrangement of collecting venules	0.14	0.03 ~ 0.61	0.0094	1.14	0.11 ~ 11.74	0.9109
Fundic gland polyp	2.59	0.22 ~ 30.98	0.4528			
Red streak	—	—	—			

^*^Odds ratio.

^**^Confidence interval.

## Discussion

The present *in vitro* study revealed that fluorescence was emitted with its activatable probe in the presence of the wild-type *H. pylori* strain, while no fluorescence was observed in the *ggt*-gene disrupted mutant. Furthermore, fluorescence emission was inhibited by the GGT-specific inhibitor. Co-localization between *H. pylori* GGT and *H. pylori* cells was observed in the stomach biopsy specimens. Together, these *H. pylori* strains produced GGT that activated gGlu-HMRG, yielding sufficient fluorescence emission via its enzymatic product, HMRG.

The present *ex vivo* imaging analysis of gGlu-HMRG revealed that a significant increase in FIVs among *H. pylori*-positive rather than -negative stomach biopsy specimens, indicating that gGlu-HMRG is applicable in diagnosing *H. pylori* infections. Furthermore, FIV differed significantly between the *H. pylori*-eradicated and *H. pylori*-positive biopsy specimens. Such pilot results suggest that gGlu-HMRG may even be useful to assess *H. pylori* eradication; however, these findings warrant further verification via larger prospective studies.

This optical fluorescence imaging method offers advantages including portability of equipment and ease of use. In the present *ex vivo* imaging assay, stomach biopsy specimens were directly treated with gGlu-HMRG, and substantial fluorescence was obtained after 5 min and more distinctly after 15 min. Current diagnostic methods for *H. pylori* infection include the rapid urease test, the ^13^C-urea breath test, anti-*H. pylori* antibody tests in body fluids, and the fecal bacterial antigen test. The most rapid diagnostic method among these diagnostic methods is still the breath analysis based on *H. pylori*-specific urease activity, yielding the *H. pylori* status in approximately 20 min in-house^16^ or in several days owing to outsourcing of the assay in actual clinical practice, even at our university hospital. However, the fluorescent method shown in the present workflow could provide results at no more than 15 min. Even at 5 min, significant differences were observed in defined FI values between *H. pylori*-positive and -negative specimens. At present, the diagnostic performance for *H. pylori* infections via *ex vivo* imaging assays of gGlu-HMRG was not superior to of other previously established diagnostic methods^16,17^. However, this fluorescence-based method may yield results more rapidly via further improvements in the assay system and methodology.

Extracellular GGT in the SHIN3 ovarian cancer cell line is potentially eliminated via rinsing with PBS when handling surgical specimens^18^. However, it is unclear whether GGT *H. pylori* is present on the cell membrane or secreted. The present results suggest that GGT is secreted into the stomach and potentially eliminated via rinsing of biopsy samples with PBS. In our *in vitro* experiment, fluorescence was observed with gGlu-HMRG in the supernatant fractions (Fig. 2), being potentially extracellular owing to secretion at least in part. Furthermore, *H. pylori* predominantly colonizes the gastric mucosa^19^, and mucus containing secreted GGT might be similarly lost during the preparation step prior to this *ex vivo* activatable probe assay. Thus, the decrease and re-localization of secreted GGT via PBS washing can affect the diagnostic performance of current *ex vivo* imaging analyses. Nevertheless, in the present study, it was necessary to rinse clinical samples obtained from an acidic environment in the stomach to neutralize pH with PBS, since gGlu-HMRG emits strong fluorescence, especially under acidic conditions. Handling of samples prior to *ex vivo* imaging with the activatable probe gGlu-HMRG remains unresolved and beyond the scope of this study. Thus, further studies are required to validate the actual performance of methods of diagnosing *H. pylori* infections with improvements in not only assay system but also preparation methodology in handling gastric samples for more accurate clinical application.

A group of gastric non-*H. pylori Helicobacter* species including *H. heilmannii*, with potential for zoonosis, are reportedly associated with mucosa-associated lymphoma in humans^20^. Urease tests are reportedly insensitive in species-level identification. Again, Pérez-Pérez *et al*. reported that urease-negative variants developed spontaneously; the frequency approached 10^−5^ from the wild-type strains of *H. pylori*^21^. A previous Japanese survey of patients with chronic active gastritis or peptic ulcers revealed 7 urease-negative *H. pylori* among the 1602 isolates^22^. Moreover, the clinically obtained urease-negative strain continued to exist for at least 42 weeks in the stomach of Mongolian gerbils, inducing gastric ulcers^22^. These data suggest that urease in *H. pylori* would not always necessarily contribute to virulence even in certain settings of such significant gastrointestinal diseases. Other diagnostic methods independent of bacterial urease activity, including the present optical fluorescence imaging assay, need to be established.

We did not perform additive sampling to investigate the association between the results of *ex vivo* gGlu-HMRG imaging and histological findings beyond the methodological protocol of the current imaging study. Sampling errors may occur during diagnosis, using biopsy-based methods of *H. pylori* infection such as the rapid urease test, despite obtaining biopsy specimens from both the antrum and stomach body. As intestinal metaplasia or atrophy may deter accurate diagnosis, we evaluated the association between *H. pylori* status assessed via the *ex vivo* gGlu-HMRG imaging assay and endoscopic findings based on the Kyoto classification of gastritis, including atrophy and intestinal metaplasia. The Kyoto classification of gastritis considers the previously established association between endoscopic findings and *H. pylori* infection status. In the classification, characteristic endoscopic findings in *H. pylori*-negative individuals are ascertained from regular sampling of collecting venules, fundic gland polyps, red streaks, and hematin, while findings such as atrophy, intestinal metaplasia, diffuse redness, enlarged fold, nodularity, xanthoma, foveolar-hyperplastic polyp, and sticky mucus were observed among individuals with *H. pylori* infections^3^. Nevertheless, the presence of an *H. pylori* infection assessed via the gGlu-HMRG *ex vivo* assay exhibited a significant correlation only with the *H. pylori* infection status. Topical application of gGlu-HMRG was useful for on-site or intraoperative identification of various cancer tissues^12,13,15,18,23,24^. Upon direct treatment of the gastric surface with gGlu-HMRG directly during endoscopic examination, it is possible to visualize *H. pylori* GGT during on-going endoscopy through fluorescence emission, independent of endoscopic findings. Further prospective studies are required to resolve sampling issues to elucidate the relationship between *ex vivo* gGlu-HMRG imaging assay and histological findings using the updated Sydney system.

## Conclusions

The gGlu-HMRG assay substantially elucidated *H. pylori* GGT activity *in vitro*. The *ex vivo H. pylori* GGT-activatable fluorescence assay is potentially applicable for rapid diagnosis of *H. pylori* infections. Further larger well-designed studies are required to validate diagnostic performance with improvements in the assay system and methodology.

## Declarations

The university ethics committees approved the study (#15012681). Ethics approval and consent to participate: Informed consent were obtained enrolled patients in this study. Consent for publication was obtained from all authors. Availability of data and material: All authors agreed to the journal guidelines.

## Supplementary information

Supplemental information
