# Supplementary material for: A novel method for rapid detection of a Helicobacter pylori infection using a γ-glutamyltranspeptidase-activatable fluorescent probe
Source: Sci Rep. 2019 Jul 1;9:9467. doi: 10.1038/s41598-019-45768-x (PMC6603024; doi:10.1038/s41598-019-45768-x)
Supplement: Supplementary file 1 — Supplemental information [file 41598_2019_45768_MOESM1_ESM.pdf]

Supplemental information

A novel method for rapid detection of a *Helicobacter pylori* infection using a γ-glutamyltranspeptidase-activatable fluorescent probe

Taro Akashi[1], Hajime Isomoto[2]*, Kayoko Matsushima[1], Mako Kamiya[3,4], Tsutomu Kanda[2], Masayuki Nakano[5,6], Takumi Onoyama[2], Masashi Fujii[2], Junko Akada[7], Yuko Akazawa[1], Ken Ohnita[1], Fuminao Takeshima[1], Kazuhiko Nakao[1], Yasuteru Urano[3,4]*

[1]Department of Gastroenterology and Hepatology, Nagasaki University Graduate School of Biomedical Sciences, 1-7-1 Sakamoto, Nagasaki 852-8501, Japan

[2]Divison of Medicine and Clinical Science, Faculty of Medicine, Tottori University, 36-1 Nishi-cho, Yonago 683-8504, Japan

[3]Graduate School of Medicine, The University of Tokyo, 7-3-1 Hongo, Bunkyo-ku, Tokyo 113-0033, Japan.

[4]Graduate School of Pharmaceutical Sciences, The University of Tokyo, 7-3-1 Hongo, Bunkyo-ku, Tokyo 113-0033, Japan

[5]Department of Bacteriology, Institute of Tropical Medicine, Nagasaki University

[6]Department of International Health, Institute of Tropical Medicine, Nagasaki University

[7]Department of Environmental and Preventive Medicine, Oita University Faculty of Medicine

The relationship between *ex vivo* gGlu-HMRG imaging assay results and extent of atrophic gastritis

	Extent of atrophy						
ex vivo gGlu-HMRG imaging assay	C-0	C-1	C-2	C-3	O-1	O-2	O-3
Antrum, FIV-15≧43.667	3	1	6	8	3	3	0
Antrum, FIV-15<43.667	11	0	2	1	2	2	0
Stomach body, FIV-15 ≧ 18.316	2	1	5	6	5	4	0
Stomach body, FIV-15<18.316	12	0	3	3	0	1	0
FI, fluorescence intensities; FIV-15, values by subtracting FI 15 minutes after sprinkling with gGlu-HMRG							
gGlu-HMRG, γ-glutamyl hydroxymethyl rhodamine green							
